# Carnitine palmitoyltransferase type 2 deficiency: novel mutation in a Native South American family with whole-body muscle magnetic resonance imaging findings: two case reports

**DOI:** 10.1186/s13256-018-1702-3

**Published:** 2018-08-28

**Authors:** Daniela Avila-Smirnow, Audrey Boutron, María de Los Ángeles Beytía-Reyes, Oscar Contreras-Olea, Alfredo Caicedo-Feijoo, Roger Gejman-Enríquez, Raúl Escobar-Henríquez, Jorge Förster-Mujica

**Affiliations:** 1 Unidad de Neurología, Servicio de Pediatría, Complejo Asistencial Dr. Sotero del Río, Avenida Concha y Toro 3459, Puente Alto, Santiago, Chile; 2 Unidad Docente Asistencial-Sótero del Río, Pontificia Universidad Católica de Chile, Santiago, Chile; 3 Unidad de Neurología, División de Pediatría, Escuela de Medicina, Pontificia Universidad Católica de Chile, Santiago, Chile; 4 Biochemistry Department, CHU Bicetre, Hôpitaux Paris-Sud, Assistance Publique – Hôpitaux de Paris, Paris, France; 5 Departamento de Radiología, Pontificia Universidad Católica de Chile, Santiago, Chile; 6 Servicio de Imagenología, Complejo Asistencial Dr. Sótero del Río, Santiago, Chile; 7 Departamento de Anatomía Patológica, Pontificia Universidad Católica de Chile, Santiago, Chile

**Keywords:** Rhabdomyolysis, Myoglobinuria, Carnitine palmitoyltransferase type II deficiency, Whole-body magnetic resonance imaging, Native South American

## Abstract

**Background:**

The myopathic form of carnitine palmitoyltransferase type II deficiency is an inherited autosomal recessive metabolic myopathy usually starting in childhood. Most reports have been on European and Japanese populations, and no Native South American patients have been reported to date. The p.Ser113Leu mutation is the most frequent in the European population. Only lower-leg magnetic resonance imaging findings have been reported, with gluteus maximus involvement in one case and normal imaging in other patients.

**Case presentation:**

Two Native South American siblings, a boy and a girl, presented to our neuromuscular clinic with recurrent rhabdomyolysis associated with transient muscle weakness after prolonged exercise. During episodes, their creatine kinase concentrations were markedly increased, up to 148,000 (1.48 × 10^5^) IU/L in the boy and 18,000 (1.8 × 10^4^) IU/L in the girl. The results of electroneuromyography and histopathology suggested a nonspecific myopathy. *CPT2* gene sequencing showed two heterozygous mutations: the p.Ser113Leu variant and a novel one (predicted to be deleterious by *in silico* analysis), the p.Ser373Pro variant. The patients’ parents were asymptomatic carriers. Whole-body magnetic resonance imaging showed mild selective involvement in the thoracic extensors and pelvic girdle in both siblings, and in the thighs and lower legs in one of them. Dietary and bezafibrate treatment was started, and symptomatic relief was observed.

**Conclusions:**

To the best of our knowledge, this is the first reported Native South American family with a CPT2 deficiency carrying a novel mutation and particular features visualized by whole-body magnetic resonance imaging.

## Background

The myopathic form of carnitine palmitoyltransferase type II (CPT II; [MIM:600650]) deficiency [MIM:255110], though a rare disease, is one of the most common causes of recurrent rhabdomyolysis and myoglobinuria in children and adults [1]. Approximately 300 cases have been reported worldwide, mainly in European and Japanese populations [2–4]. The frequency of CPT II deficiency has probably been underestimated, owing to low clinical suspicion and difficulty in performing confirmatory laboratory tests. Particularly in South American populations and in patients belonging to Native South American ancestry groups, no previous reports are found. CPT II deficiency is inherited in an autosomal recessive manner. The *CPT2* gene is located in chromosome 1p.32.3 and codes for an inner mitochondrial membrane protein. Its main function is the transesterification of long-chain fatty acids that allows its transport to the inner mitochondria, where β-oxidation takes place. Therefore, CPT II deficiency is considered a β-oxidation defect [5].

There are three different phenotypic forms of CPT II deficiency. The myopathic form, the most frequent and milder of the phenotypes, is clinically characterized by recurrent rhabdomyolysis starting in childhood, triggered by prolonged physical exercise, fasting, or viral infections [3]. The diagnostic workup should include a complete neurological examination, muscle biopsy with Oil Red O stain, and acylcarnitine profile (increased long-chain fatty acylcarnitine). Enzymatic testing is usually helpful, and genetic testing is confirmatory. Lower-leg muscle magnetic resonance imaging findings in one patient with CPT II deficiency showed mild involvement in T1-weighted sequences in pelvic girdle muscles [6].

Symptomatic treatment, including avoiding prolonged exercise and diet modifications, is recommended. Bezafibrate, a hypolipidemic drug, showed beneficial effects, reducing muscle pain and increasing physical activity in a long-term clinical trial [7]. Later, a 3-month randomized controlled trial did not demonstrate significant effects in fatty acid oxidation and heart rate [8]. So, bezafibrate therapy remains controversial. In this report, we present a Chilean Native South American family with a myopathic form of CPT II deficiency, a novel genetic mutation, and a particular involvement as determined by muscle whole-body magnetic resonance imaging (WBMRI).

## Case presentation

A descriptive, retrospective medical record review was performed after Institutional Review Board approval was obtained (local ethics committee: Comité Etico Científico Servicio Salud Metropolitano Sur Oriente). Two siblings from a nonconsanguineous family with a Native South American ethnic background presented to our neuromuscular clinic. Both parents belong to the Mapuche ethnic group and were from the Araucanía region in Chile. The mother was a healthy young woman; the father had had cerebral palsy since birth (hemiparesis and mild cognitive delay). The family history was otherwise irrelevant. Pregnancy, childbirth, and psychomotor development were normal in both children.

The brother (patient 1), a 20-year-old man, had had recurrent episodes of rhabdomyolysis after prolonged physical exercise since age 4 years. He presented to our clinic at age 16 years. The results of his physical neurological examinations, including muscle strength testing, were completely normal during the first years of follow-up. At the age of 19 years, permanent iliopsoas mild muscle weakness was found. The sister (patient 2), a 13-year-old girl, presented with similar episodes of rhabdomyolysis with a milder severity, starting at age 8 years, with normal muscle strength. Electroneuromyography showed myopathic signs in both patients.

During rhabdomyolysis, the brother’s creatine kinase (CK) reached 148,000 (1.48 × 10^5^) IU/L (reference value [RV], 0–190 IU/L), and he had a normal acylcarinitine profile (acetylcarnitine, propionylcarinitine, butyrylcarnitine, isovalerylcarnitine, octanoylcarinitine, myristoylcarnitine, palmitoylcarinitine). The sister’s CK levels during rhabdomyolysis reached 18,000 (1.8 × 10^4^) IU/L. At baseline, her CK was 65 IU/L, acylcarnitine profile was normal, total carnitine was 69 µmol/L (RV, 36–56 µmol/L), free carnitine was 37 µmol/L (RV, 19–35 µmol/L), and acylcarnitine was 32 µmol/L (RV, 4–14 µmol/L). The results of cardiologic, respiratory, and renal evaluations were normal in both patients.

### Histopathology

Muscle biopsies were performed in both siblings (H&E, NADH and ATPase stains). The brother had mild endomysium enlargement, a few central nuclei, and type II fiber predominance. The sister had mild endomysium enlargement, type I fiber predominance, a few mildly atrophic type II fibers, and some fibers with mild increases in subsarcolemmal basophilic material. The results of electron microscopy were normal in both siblings. A nonspecific myopathy was determined in both siblings.

### Mutation analysis of *CPT2* gene

After informed consent was obtained, DNA was collected from the two siblings and their parents and extracted from whole blood using the DNeasy Mini Kit (QIAGEN, Hilden, Germany). Five pairs of primers were designed to amplify five fragments of the *CPT2* gene (RefSeq: NM_000098.2), covering each exon and their respective splice site junctions. PCR products were labeled with the Applied Biosystems BigDye terminator version 1.1 sequencing kit (Thermo Fisher Scientific, Waltham, MA, USA) and purified with the DyeEx2.0 spin kit (QIAGEN). The sequence products were run on an automated ABI 3130XL gene analyzer (Thermo Fisher Scientific), and the results were analyzed with SeqScape 2.6 software (Thermo Fisher Scientific). The primer sequences are available upon request from the corresponding author. *In silico* analysis of missplicing effects was achieved using Alamut version 2.3 integrated software (Interactive Biosoftware, Rouen, France; http://www.interactive-biosoftware.com/). One novel mutation (c.1117T>C [exon 4] p.Ser373Pro) predicted to be damaging (Align DGVD, Polyphen-2, SIFT, MutationTaster) inherited from the father and a previously reported missense mutation (c.338C>T [exon 3], p.Ser113Leu) inherited from the mother were identified in the *CPT2* gene. The variant was published in the Leiden Open Variation Database (http://www.lovd.nl).

### Whole-body magnetic resonance imaging

**Fig. 1:**
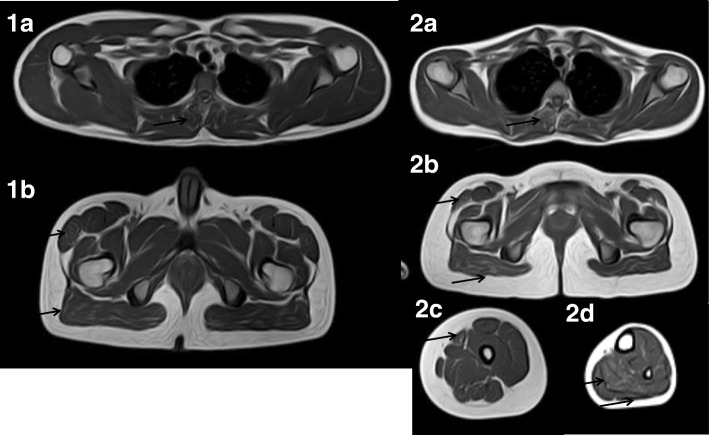
Whole-body magnetic resonance imaging T1-weighted axial sections in both siblings (1a and 1b: boy; 2a–2d: girl). Involved muscles are depicted by *black arrows*. 1a and 2a: Mild hypersignal of thoracic extensors. 1b and 2b: Mild hypersignal of tensor fasciae latae and gluteus maximus. 2c: Sartorius mild hypersignal. 2d: Soleus mild hypersignal and medial gastrocnemius atrophy

WBMRI with coronal and axial short tau inversion recovery (STIR) and T1-weighted sequences was performed using a 1.5-T Achieva scanner (Philips, Eindhoven, the Netherlands). The STIR sequences were normal. On T1-weighted images, the head, neck, and upper limb muscles were speared in both patients. Both siblings had selective mild involvement of thoracic the extensors, the tensor fasciae latae, and the gluteus maximus. The sister also had sartorius and soleus mild involvement and marked atrophy of the lateral gastrocnemius (Fig. 1).

### Treatment and follow-up

Diet and bezafibrate treatment was advised. In the brother, symptomatic relief was observed (muscle pain reduction); he finished high school and is currently a baker. The sister has not yet started pharmacologic treatment; she normally attends school and practices sports with some limitations.

## Discussion and conclusions

We report a family with a Native South American ethnic background, belonging to the Mapuche ethnic group, with a myopathic form of CPT II deficiency. To the best of our knowledge, these are the first cases of CPT II deficiency reported in the literature in patients with a Native South American genetic background. The clinical phenotype in our patients is characteristic of this form of CPT II deficiency, starting at childhood and presenting as recurrent rhabdomyolysis with electroneuromyographic and histopathologic findings showing a nonspecific myopathy. The clinical picture is more severe in the brother than in the sister, probably owing to males usually performing more intense and prolonged physical exercise.

A heterozygous compound *CPT2* gene mutation was found in both siblings. One of their mutations, the p.Ser113Leu variant, is the most frequent (60%) in white populations. The other one, p.Ser373Pro, segregating with the disease and predicted to be deleterious by *in silico* analysis, is a novel one.

Muscle magnetic resonance imaging in metabolic myopathies or pseudometabolic myopathies has been well documented in Pompe disease [9]. Only a few reports of exercise intolerance and recurrent rhabdomyolysis have been published [6, 10, 11]. In 2015, lower limb muscle magnetic resonance imaging findings in a series of 14 patients with long-chain fatty acid oxidation metabolic myopathies were reported [6]. Three patients with CPT II deficiency were included, two of whom had a normal pattern and one of whom had mild gluteus medius and maximus involvement in T1-weighted images. In our patients, we observed T1-weighted thoracic extensor involvement in both patients. The sister also had thigh and lower-leg involvement. STIR sections and imaging of other body regions were normal. We think that WBMRI might be a useful diagnostic tool in recurrent rhabdomyolysis, contributing to differentiation of particular entities such as other β-oxidation defects, glycogen storage diseases, and muscular dystrophies [12].

This is one of the few inherited myopathies with specific treatment, because bezafibrate has been an effective therapeutic option in patients with CPT II deficiency, and in one of our patients, treatment was indeed associated with muscle pain reduction. We highlight the relevance of early diagnosis in order to provide appropriate treatment, avoid exacerbations, and prevent renal failure.

## Abbreviations

CK: Creatine kinase
CPT II: Carnitine palmitoyltransferase type II
RV: Reference value
STIR: Short tau inversion recovery
WBMRI: Whole-body magnetic resonance imaging

## Authors’ information

DAS is a pediatric neurologist and neuromuscular disease specialist at Sótero del Río Hospital and Pontificia Universidad Católica de Chile.
